# Prevalence and Types of Otological Symptoms Presenting in COVID-19 Patients in Jeddah, Saudi Arabia: A Questionnaire-Based Study

**DOI:** 10.7759/cureus.42042

**Published:** 2023-07-17

**Authors:** Hosam Amoodi, Osamah Abualross, Nuha Meer, Nadin A Alharbi

**Affiliations:** 1 Department of Otolaryngology, Head and Neck Surgery, University of Jeddah, Jeddah, SAU; 2 Department of Otolaryngology, Head and Neck Surgery, Dr. Soliman Fakeeh Hospital, Jeddah, SAU; 3 Faculty of Medicine, University of Jeddah, Jeddah, SAU; 4 Faculty of Medicine, King Abdulaziz University, Jeddah, SAU

**Keywords:** saudi arabia, imbalance, vertigo, dizziness, otological manifestations, ear pain, covid-19

## Abstract

Background

The clinical manifestations of coronavirus disease 2019 (COVID-19) can cause sensory dysfunction of taste, smell, and hearing. Otological symptoms may exceed hearing loss to ear pressure, tinnitus, and hyperacusis.

Objective

The objective of this study was to identify the prevalence and types of otological symptoms among patients diagnosed with COVID-19 in Jeddah, Saudi Arabia.

Methods

This is a cross-sectional study that was conducted among COVID-19 patients who have been diagnosed at Dr. Soliman Fakeeh Hospital (DSFH), Jeddah KSA, aged 18 years or older. The data collection was done through phone-call interviews utilizing an online form of a pre-structured questionnaire. The form included six otological symptoms; each symptom was further detailed with regard to duration, intensity, clinical course (continuous vs intermittent), and recovery.

Results

A total of 406 responses from patients diagnosed with COVID-19 were analyzed. Females represented 53.7% of the sample. The highest proportion of patients (30%) was in the age group of 31-40, followed by 22.9% in the age group of 25-30. The otological symptoms' prevalence rates were as follows: dizziness, vertigo, or imbalance 34.5%, ear pain 13.1%, tinnitus 12.1%, ear pressure 10%, hearing loss 6.4%, and hyperacusis 5.4%. Males had a higher prevalence of tinnitus, while females had higher reported symptoms of ear pain, hearing loss, and hyperacusis.

Conclusion

The most common otological symptoms were dizziness, vertigo, and imbalance among one-third of COVID-19 patients. Females reported higher rates of symptoms with ear pressure having significantly higher odds among females. Age groups were also significantly associated with ear pain, tinnitus, and ear pressure.

## Introduction

Coronavirus disease 2019 (COVID-19) which is caused by the virus SARS-CoV-2 is a large positive-stranded, enveloped non-segmented RNA virus [1,2]. On the 6th of January 2020, COVID-19 was identified as the cause of several unidentified pneumonia cases reported in Wuhan, China. Since then, the World Health Organization (WHO) has declared a pandemic state due to the rapid spread of the virus to over 200 countries and territories [3].

The clinical course of COVID-19 can range from being asymptomatic to the development of severe acute respiratory distress, which can lead to multiorgan involvement and ultimately death [4,5]. The typical and most widely agreed upon manifestations of a confirmed COVID-19 case are usually mild including fever, fatigue, headache, myalgia or arthralgia, chills, dry cough, sputum production, shortness of breath, sore throat, and gastrointestinal disturbances [5-8]. COVID-19 has proven to be highly infectious with person-to-person transmission occurring despite the absence of clinical symptoms [6,7].

The effect of COVID-19 on extrapulmonary systems such as the nervous systems has been established in the literature, primarily the gustatory, olfactory, and visual involvement [9-14]. Viruses have long been proven to affect hearing and cause ear-related symptoms through direct damage to inner ear structures or immune-mediated damage, yet they seem to be often neglected in clinical practice [1,15]. Multiple systematic reviews showed a statistically significant association between otological symptoms (hearing loss, dizziness, tinnitus) and COVID-19 disease [1,5]. An Italian study surveying a total of 185 confirmed COVID-19 patients from 15 different hospitals showed that 43 (23.2%) had tinnitus, 34 (18.4%) experienced disequilibrium, two of which reported acute attacks of vertigo and 32 showed dizziness, while 14 (7.6%) reported both tinnitus and disequilibrium [16]. On the other hand, another study demonstrated that the audio-vestibular symptoms of COVID-19 are mostly minor, few, and uncommon with an incidence rate of approximately less than 1% [3].

As indicated by multiple studies, little attention is being placed on the impact of COVID-19 on the auditory-vestibular system. More investigations focusing on the otological symptoms of COVID-19 need to be carried out [1,3,17]. As the diagnosis of COVID-19 is mainly suspected among patients with typical pulmonary symptoms, missed cases can contribute to an increased rate of infection transmission [5].

As patients suffering from audio-vestibular symptoms have an increased risk of permanent complications without being diagnosed or managed properly, more attention should be given to audio-vestibular symptoms and their role in COVID-19 patients. The ignorance of otological symptoms is mainly due to a lack of literature and knowledge on the relationship between COVID-19 and otological manifestations. Therefore, this study aimed to identify the prevalence and types of otological symptoms among patients diagnosed with COVID-19 at Dr. Soliman Fakeeh Hospital, Jeddah, Saudi Arabia.

## Materials and methods

Study design

A cross-sectional study was conducted at Dr.Soliman Fakeeh Hospital (DSFH), tertiary center, Jeddah, Saudi Arabia, from May 2022 to October 2022 in the Otolaryngology-Head and Neck Surgery Department.

Study population

A total of 406 adult COVID-19 patients of both sexes, aged 18 and above, who have had a confirmed COVID-19 diagnosis by PCR test or rapid antigen test at DSFH, were included using simple random sampling. Hospitalized patients, patients with pre-existing otological pathology or who underwent ear surgery, patients treated with chloroquine and hydroxychloroquine, and those who received oxygen therapy were excluded.

The sample size was calculated using an online calculator for cross-sectional studies (raosoft.com) [18]. The equation was built using a 5% margin of error, 95% confidence level, and 50% distribution rate. The calculated sample size was 385 patients.

Data collection

The data collection was done through patient interviews via phone calls utilizing a pre-structured questionnaire. This questionnaire has an Arabic and English version, and both had been validated using face validation by ENT consultants and specialists at DSFH. The questionnaire included three sections. The first section inquired about patients' demographic characteristics including age, gender, nationality, and comorbidities. The second section inquired about COVID-19 history and vaccination. The third section included six otological symptoms: hearing loss, ear pain, tinnitus, ear pressure, hyperacusis, and disequilibrium (dizziness, vertigo, or imbalance). Each symptom of the third section was further detailed in terms of duration, intensity, clinical course (continuous vs intermittent), and recovery.

Data analysis

IBM SPSS Statistics for Windows, Version 29 (Released 2021; IBM Corp., Armonk, New York, United States) was used. Frequency and proportions were used to summarize categorical variables. Mean and standard deviation, median, and interquartile range were used to summarize continuous variables as appropriate according to the Shapiro-Wilk normality distribution test. The chi-square test and Fisher-Freeman-Halton exact test were used to test associations between categorical variables. The significance level was set at (0.05).

Ethical considerations

The current study was approved by the research and ethics committee of Dr. Soliman Fakeeh Hospital, Jeddah, Saudi Arabia (273/IRB/2022). All the collected data were handled with high confidentiality to maintain participants' privacy and were used for research purposes only.

## Results

A total of 406 responses from patients diagnosed with COVID-19 were analyzed. Females represented 53.7% of the sample. The highest proportion of patients (30%) was in the age group of 31-40, followed by 22.9% in the age group of 25-30. Hypertension was the most common comorbidity (15.3%). The sociodemographic variables and comorbidities are shown in Table 1. The majority (68.2%) had three doses at the time of infection. One-quarter of the patients (26.1%) were diagnosed with COVID-19 more than once. The COVID-19 vaccination details are shown in Table 2.

**Table 1:** Sociodemographic characteristics and comorbidities of the patients

Variable	Groups	Count	Column N %
Age	18-24	55	13.5%
	25-30	93	22.9%
	31-40	122	30%
	41-60	76	18.7%
	Above 60	60	14.8%
Gender	Male	188	46.3%
	Female	218	53.7%
Nationality	Non-Saudi	93	22.9%
	Saudi	313	77.1%
Comorbidity	Diabetes mellitus (DM)	33	8.1%
	Hypertension (HTN)	62	15.3%
	Hyperlipidemia	37	9.1%
	Asthma	13	3.2%

**Table 2:** Vaccination and COVID-19 status of the patients

		Count	Column N %
Vaccine status at the time of infection	One dose	16	3.9%
	Two doses	80	19.7%
	Three doses	277	68.2%
	More than 3	11	2.7%
	None	22	5.4%
Have you had COVID-19 more than once?	Yes	106	26.1%
	No	300	73.9%

The otological symptoms prevalence rates were as follows: dizziness, vertigo, or imbalance 34.5%, ear pain 13.1%, tinnitus 12.1%, ear pressure 10%, hearing loss 6.4%, and hyperacusis 5.4%.

Symptom improvement was inquired and there was improvement in the majority of symptoms except for hyperacusis which only improved among half of the affected patients (50%). Details about the affected ears, duration of symptoms, course, and improvement are shown in Table 3.

**Table 3:** Otological symptoms and their characteristics among the patients

	Hearing loss	Pain	Tinnitus	Pressure	Hyperacusis	Dizziness etc.
	N=26	N=53	N=49	N=43	N=22	N=140
Affected ear						
Right	5 (19.2%)	18 (34%)	19 (38.8%)	6 (14%)	4 (18.2%)	N/A
Left	4 (15.4%)	14 (26.4%)	12 (24.5%)	4 (9.3%)	2 (9.1%)	N/A
Both	17 (65.4%)	21 (39.6%)	18 (36.7%)	33 (76.7%)	16 (72.7%)	N/A
Duration						
Less than a week	9 (34.6%)	23 (43.4%)	22 (44.9%)	26 (60.5%)	6 (27.3%)	N/A
1-4 weeks	17 (65.4%)	19 (35.8%)	19 (38.8%)	9 (20.9%)	6 (27.3%)	N/A
More than four weeks	0 (0%)	11 (20.8%)	8 (16.3%)	8 (18.6%)	10 (45.5%)	N/A
Course						
Continuous	18 (69.2%)	6 (33.3%)	17 (34.7%)	24 (52.5%)	8 (36.4%)	N/A
Intermittent	8 (30.8%)	12 (66.7%)	32 (65.3%)	20 (46.5%)	14 (63.6%)	N/A
Improved						
Yes	14 (53.8%)	15 (83.3%)	37 (75.5%)	35 (81.4%)	11 (50%)	123 (87.9%)
No	12 (46.2%)	3 (16.7%)	12 (24.5%)	8 (18.6%)	11 (50%)	17 (12.1%)

**Figure 1:**
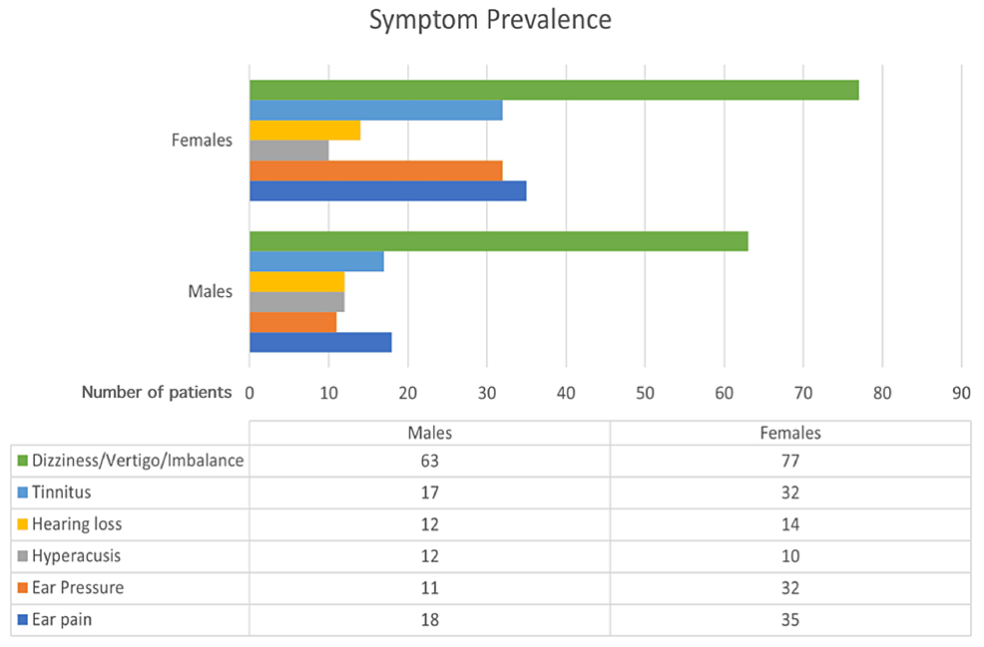
Comparison of the distribution of the otological symptoms between males and females

Of those who had otological symptoms (181 out of 406, 44.6%), only 12.7% consulted a physician. The presence of each otological symptom was further tested for significant association with gender and age groups. Generally, females had a higher prevalence of symptoms except for tinnitus. Furthermore, those who reported ear pressure had significantly higher odds of being females (OR=2.8, P-value=0.004), while other symptoms did not show any significance with gender. The frequency of symptoms is demonstrated by gender in Figure 1.

**Figure 2:**
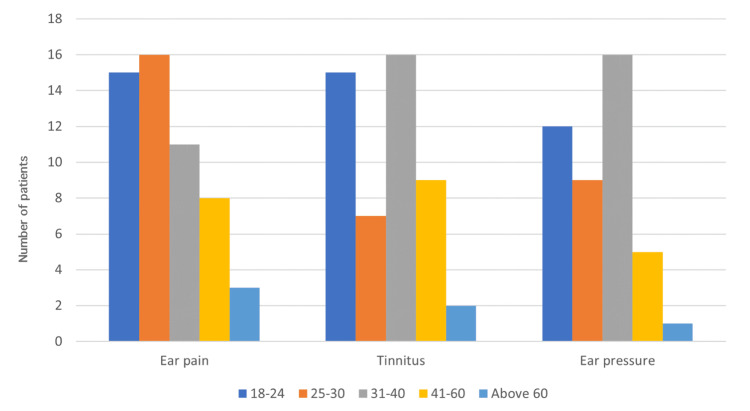
The distribution of significant symptoms among age categories

Age showed a significant association with ear pain (P-value=0.002), tinnitus (P-value=0.001), and ear pressure (P-value=0.006). The demonstration of significant symptoms across age groups is shown in Figure 2. Further analyses of dizziness, vertigo, and/or imbalance with age, gender, and comorbidities did not show statistical significance.

## Discussion

Although multiple studies had showed a significant association between COVID-19 and otological symptoms, there are deviations in the literature regarding this topic. This study aims to determine if there is a significant relationship between COVID-19 and otological symptoms. Reported symptoms included hearing loss, hyperacusis, ear pressure, dizziness, and tinnitus [1,5]. This is evident from previous reports which indicate that a significant association between COVID-19 and newly diagnosed otological conditions such as hearing loss, dizziness, and tinnitus has been previously reported [6]. Otological symptoms among COVID-19 patients should not be considered just as parallel manifestations to the main disease. On rare occasions, COVID-19 patients reported sudden sensorineural hearing loss as the only presenting symptom [19]. While otological manifestations can be described as atypical signs of COVID-19, it has been reported that hearing loss and tinnitus were persistent symptoms even after the resolution of the main symptoms of COVID-19 in 50% and 25%, respectively [20,21]. While the exact cause of some vestibular symptoms is not fully described in the literature, it has been suggested that mild vestibular symptoms such as dizziness and imbalance might be a result of the profound fatigue and asthenia that are usually experienced by COVID-19 patients [21]. Another opinion regarding hearing loss and balance disorders might be due to the vascular damage of the inner ear structures that are particularly susceptible to ischemia because of their characteristics of terminal vasculature and high-energy requirements [22].

In the present study, the prevalence rates of dizziness, tinnitus, and hearing loss were 34.5%, 12.1%, and 6.4%, respectively. When comparing these results to the literature, a recently published meta-analysis of 12 studies pointed some discrepancies showing the occurrence rates of hearing loss (3.1%), tinnitus (4.5%), and dizziness (12.2%) with a pooled total prevalence of 7.6% [5]. This discrepancy can be explained by the fact that minor attention would be paid to otological symptoms in a respiratory viral infection with complete ignorance of mild otological symptoms among severe cases of COVID-19 [5]. The study design and tools of measurement can play a role in making a discrepancy in the prevalence rates. Subjective self-reports would raise the prevalence rate and show exaggerated severity compared to objective otological assessment [23,24]. In addition, we found a significant association between gender and the presence of otological symptoms, which was found to be congruent with a recent article showing that the otological symptoms were more frequent in females, with a female: male ratio of 2.3:1 [25]. Moreover, in parallel to the literature, our findings showed another significant association of the presence of otological symptoms including ear pain, tinnitus, and ear pressure with age [26].

With the discussion of our study findings, its limitations should be pointed out when interpreting the findings. First, neither temporality nor causation can be determined in a cross-section design, which can be considered a limitation in our study. Moreover, given the subjective nature of data collection, this study also provides little evidence considering the severity of otological symptoms (i.e., mild to moderate) and the type of hearing loss (i.e., sensory-neural hearing loss, conductive hearing loss). On the other hand, and to our knowledge, studies regarding otological manifestations of COVID-19 vaccination are rare, which can be determined as a strength of this study. Moreover, the present study can indicate the impact of COVID-19 disease and post-vaccination on the occurrence of otological symptoms to better understand the burden of the disease. Nevertheless, we can confidently say that this study is significantly important and adds to the literature.

## Conclusions

There is no doubt that COVID-19 affects the respiratory tract primarily. However, along with other studies, this study proves the otological manifestation of COVID-19. To sum up, the obtained data in this study indicate a higher prevalence of otological symptoms among COVID-19 patients (44.6%) of whom the majority were fully vaccinated (68.2%). In contrast, the most common otological symptoms were dizziness followed by ear pain, tinnitus, ear pressure, and hearing loss. Our findings also revealed a significant association of the presence of otological symptoms with gender and age. Conducting additional studies nationally, with larger sample sizes, and more objective data, in addition to clinical assessments, is recommended for further understanding the otological manifestation of COVID-19 vaccination.
